# Acute and Subacute Toxicity Studies of *Erodium guttatum* Extracts by Oral Administration in Rodents

**DOI:** 10.3390/toxins14110735

**Published:** 2022-10-27

**Authors:** Kaoutar Benrahou, Hanae Naceiri Mrabti, Hamza M. Assaggaf, Salma Mortada, Najoua Salhi, Lamiaa Rouas, Rim El Bacha, Abdellah Dami, Azlarab Masrar, Mohammed Merae Alshahrani, Ahmed Abdullah Al Awadh, Abdelhakim Bouyahya, Khang Wen Goh, Long Chiau Ming, Yahia Cherrah, My El Abbes Faouzi

**Affiliations:** 1 Laboratory of Pharmacology and Toxicology, Bio Pharmaceutical and Toxicological Analyzes Research Team, Faculty of Medicine and Pharmacy, Mohammed V University in Rabat, Rabat 10100, Morocco; 2 Department of Laboratory Medicine, Faculty of Applied Medical Sciences, Umm Al-Qura University, Makkah 21955, Saudi Arabia; 3 UPR of Pathological Anatomy and Cytology, Faculty of Medicine and Pharmacy, Mohammed V University in Rabat, Rabat 10100, Morocco; 4 Military Instruction Hospital Mohammed V, Rabat 10045, Morocco; 5 Central Laboratory of Hematology, Ibn Sina Hospital, Faculty of Medicine and Pharmacy, Mohammed V University in Rabat, Rabat 10045, Morocco; 6 Department of Clinical Laboratory Sciences, Faculty of Applied Medical Sciences, Najran University, Najran 61441, Saudi Arabia; 7 Laboratory of Human Pathologies Biology, Department of Biology, Faculty of Sciences, and Genomic Center of Human Pathologies, Mohammed V University in Rabat, Rabat 10106, Morocco; 8 Faculty of Data Science and Information Technology, INTI International University, Nilai 71800, Malaysia; 9 Pengiran Anak Puteri Rashidah Sa’adatul Bolkiah Institute of Health Sciences, Universiti Brunei Darussalam, Gadong BE1410, Brunéi

**Keywords:** *Erodium guttatum*, acute toxicity, subacute toxicity, safety, histopathology

## Abstract

The present study aimed to evaluate the acute and subacute toxicity profiles of *Erodium guttatum* extracts in mice using the methods described in the guidelines of the OECD. In the acute toxicity study, the LD_50_ value was greater than 2000 mg/kg. The subacute toxicity study of *E. guttatum* extracts showed no significant changes in body or organ weights. The administration of *E. guttatum* extracts to mice at a dose of 200 mg/kg led to an increase in white blood cells, platelets and hemoglobin. Moreover, the aqueous extract of *E. guttatum* only decreased liver aspartate aminotransferase (ASAT) levels at a dose of 200 mg/kg, and creatinine and urea levels did not show any significant alterations compared to the control group. Our results showed that the extracts of *E. guttatum* caused a slight increase in alanine aminotransferase (ALAT) and triglycerides. The histological study showed that mice treated with *E. guttatum* extracts experienced some histopathological changes in the liver, particularly with the methanolic extract, and slight changes in the kidneys and pancreas. Regarding the renal profile, no toxicity was observed. These results provide basic information on the toxicological profile of *E. guttatum* used in traditional medicine.

## 1. Introduction

For a long time, natural resources have been used as remedies to prevent or cure various diseases due to their powerful therapeutic effects and low costs. People consume natural products because they think they are safe and do not have harmful effects [1]. Medicinal plants can contain many secondary metabolites, some of which are very complex. Nevertheless, herbs used to treat certain diseases are generally used without any scientific knowledge or evidence of toxicological effects [2,3].

The acute and subacute toxicity test is a method based on an evaluation of the harmlessness and safety of chemicals, as well as an analysis of their mode of action. Acute and subacute systemic toxicity studies are used for hazard disclosure and risk management in the context of the production, handling and use of chemicals [4]. Acute toxicity is a single-dose test that identifies symptoms and the extent to which toxicity affects animals [5]. Subacute toxicity studies at repeated doses can be carried out after obtaining preliminary information from acute toxicity tests, which provide information about the animal’s target tissue or organ [6].

The genus *Erodium* of the Geraniaceae family includes more than 70 species distributed in all continents, of which 63 are concentrated in the Mediterranean regions [7,8]. Indeed, only seven species do not occur in Europe or the Mediterranean region. Of these, one is in Central Asia, one in East Asia, one in South Africa, two in western North America, one in South America and one in Australia [9]. In addition, 16 of them have been reported as endemic to Turkey [10]. *Erodium* species are traditionally used to prepare astringent and antiseptic teas. Decoctions of the aerial parts are used as a remedy for dysentery, fever, wounds and worm infections. The leaves have been used in the kitchen for the preparation of salads, omelets, sandwiches, sauces and soups and certain food products [11,12]. *Erodium* is used to treat and/or prevent dermatological and gastrointestinal pathologies, indigestion and inflammatory diseases, diabetes, cancer, constipation, eczema, hemorrhage, and as a carminative, astringent and antiseptic agent [13]. *Erodium* is characterized by five fertile and five infertile flowers, which are regular or zygomorphic and glandular. The leaves are mostly pinnate or undivided (pinnatifid/pinnatisect). Leaf size and shape can vary between populations, and the variation is usually pronounced within populations [14].

The species *Erodium guttatum* (Desf.) Willd. is a perennial plant found in North Africa, southern Spain and Palestine. In Morocco, it is known as "Wedmi." Pharmacological studies have indicated that it has antioxidant and antimicrobial abilities [15]. To the best of our knowledge, no chemical or toxicological studies have been performed on *Erodium guttatum* roots. A safety evaluation of this herb is needed. In this respect, the objective of this study was to evaluate the possible acute and subacute toxic effects of the root extracts of the species *E. guttatum*.

## 2. Results

### 2.1. Acute Oral Toxicity

Oral administration of aqueous, ethanolic and methanolic extracts of *E. guttatum* at a dose of 2 g/kg showed no mortality in treated mice. Treatment with each extract did not induce weight loss, and no behavioral disorder was recorded during the 14 days of observation. Therefore, the oral LD_50_ of *E. guttatum* is greater than 2000 mg/kg (Table 1).

### 2.2. Subacute Oral Toxicity

#### 2.2.1. Body Weights

The groups of mice treated with the three extracts of *E. guttatum* at a dose of 200 mg/kg recorded no significant change (*p* > 0.05) in body weights compared to the control group (Table 2).

#### 2.2.2. Organ Weights

The resulting organ weights are shown in Table 3. Macroscopic analysis of the target organs (liver, kidney and pancreas) of mice treated with aqueous, ethanolic and methanolic extracts of *E. guttatum* showed there were no significant changes in color or texture compared to the control group.

#### 2.2.3. Hematological Parameters

The hematological results are summarized in Table 4. The animals treated with the aqueous, ethanolic and methanolic extracts of *E. guttatum* showed elevated levels of white blood cells (WBC), red blood cells (RBC), platelets (PLT), lymphocytes (LYMPH), monocytes (MONO), basophils (BASO) and neutrophils (NEUT) compared to the control. Similarly, the administration of the ethanolic and methanolic extract at a dose of 200 mg/kg caused no significant difference (*p* > 0.05) in the levels of hematological parameters.

#### 2.2.4. Serum Biochemical Parameters

The results of the biochemical parameters are shown in Table 5. Daily oral administration of *E. guttatum* extracts to mice in all groups caused a slight increase in cholesterol and low-density lipoprotein (LDL) compared with mice in the control group.

Regarding the levels of aspartate aminotransferase (ASAT), there was a non-significant decrease observed in the group of mice treated with the aqueous extract compared to the control group, while there was an increase in ASAT levels in the groups of mice treated with methanolic and ethanolic extracts. Similarly, the levels of alanine aminotransferase (ALAT) in the group of control mice and the group of mice treated with aqueous extract were equal. It can be seen that the mice treated with methanolic extract had higher levels of ASAT and ALAT compared to the groups of mice treated with ethanolic and aqueous extracts.

Regarding urea levels, there was a non-significant decrease in all groups of mice treated with the three extracts of *E. guttatum* compared to the control mice. For creatinine levels, there was an increase in the groups of mice treated with the ethanolic and methanolic extracts and a decrease in the group of mice treated with the aqueous extract.

#### 2.2.5. Histopathology

**Figure 1:**
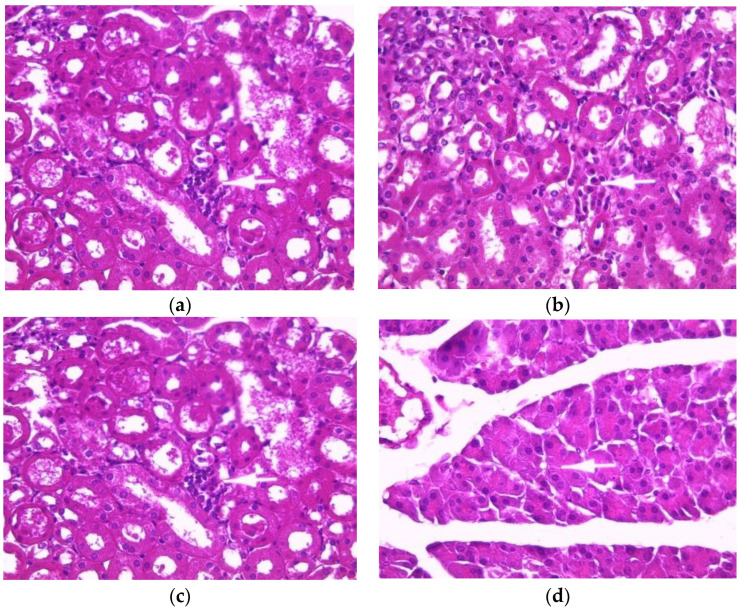
Effect of *E. guttatum* extracts on renal histology in mice. Histological sections were visualized by hematoxylin and eosin (H and E) staining and observed under a light microscope (OPTIKA Microscopes, Italy) at 40× magnification. (**a**) Control mice, (**b**) mice treated with aqueous extract of *E. guttatum* (200 mg/kg), (**c**) mice treated with ethanolic extract of *E. guttatum* and (**d**) mice treated with methanolic extract of *E. guttatum*.

Normal histology of mouse kidneys (glomeruli, tubules and interstitium) was found in the control group (Figure 1a). Mice treated with an aqueous extract (200 mg/kg) of *E. guttatum* showed minimal interstitial inflammation (Figure 1b). These observations corroborate the results of serum analysis. The histology of kidney sections from mice treated with ethanolic extract revealed minor or mild changes, including focal interstitial inflammation and increased plasma cells (Figure 1c). Conversely, the histology of kidney sections from mice treated with the methanolic extract showed major abnormalities: glomerular injury, severe interstitial inflammation and plasma cells in the interstitium (Figure 1d).

**Figure 2:**
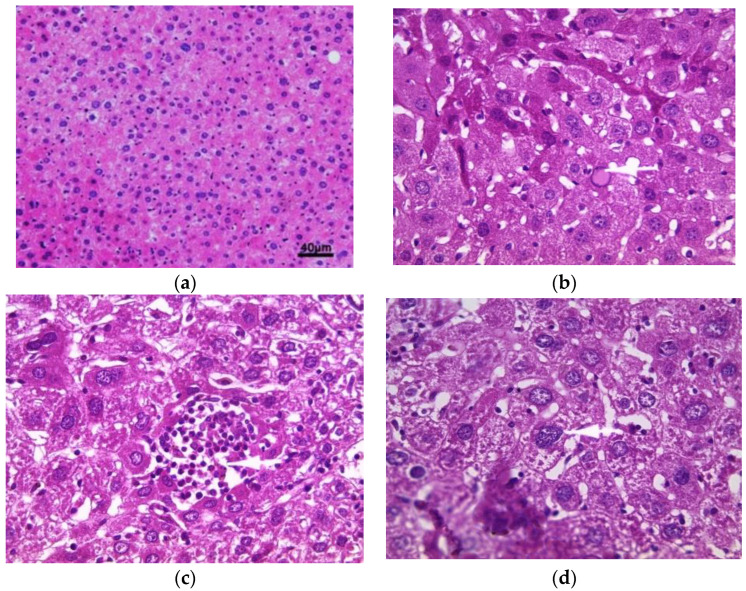
Histopathology of the liver. Histological sections were visualized by hematoxylin and eosin (H and E) staining and observed under a light microscope (OPTIKA Microscopes, Italy) at 40× magnification. (**a**) Control mice, (**b**) mice treated with aqueous extract of *E. guttatum* (200 mg/kg), (**c**) mice treated with ethanolic extract of *E. guttatum* and (**d**) mice treated with methanolic extract of *E. guttatum*.

The histology of liver sections from the control mice showed normal hepatocellular architecture as well as well-preserved liver cells, visible central veins and no histological abnormalities (Figure 2a). The liver sections from the aqueous extract (200 mg/kg)-treated groups showed some histological changes, such as intralobular inflammation around the centrilobular veins (CLVs) and cytoplasmic hydropic degeneration of hepatocytes (Figure 2b). In contrast, subacute administration of ethanolic extract caused minimal intralobular inflammation and hydropic degeneration of the cytoplasm, showing binucleated cells and a somewhat enlarged nucleus with blackish pigments (Figure 2c). The liver sections of mice treated with methanolic extract showed an intralobular mononuclear inflammatory focus around the centrilobular veins, hepatocytes with hyperchromatic nuclei, moderate binucleation, vacuolated and enlarged nucleoli, predominant nucleoli (one or two), multinucleated hepatocytes, blackish pigments, and intra sinusoidal (Figure 2d).

**Figure 3:**
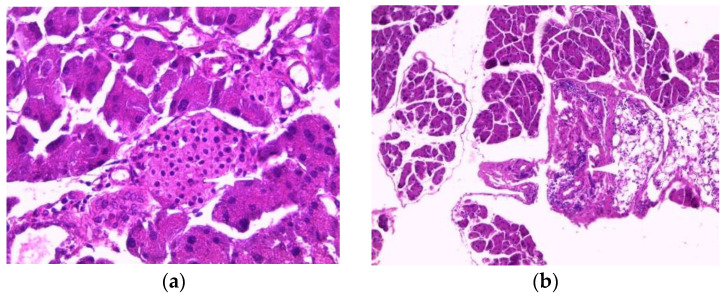

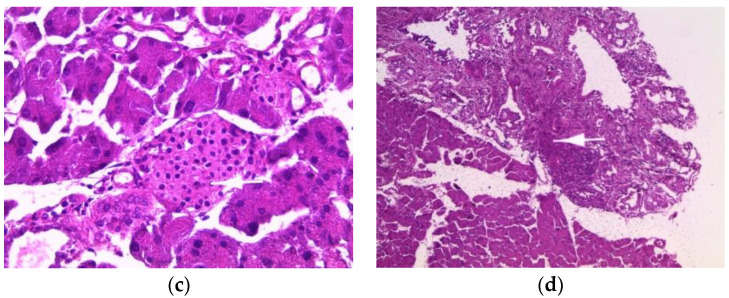
Histopathology of the pancreas. Histological sections were visualized by hematoxylin and eosin (H and E) staining and observed under a light microscope (OPTIKA Microscopes, Italy) at 40× magnification. (**a**) Control mice, (**b**) mice treated with aqueous extract of *E. guttatum* (200 mg/kg), (**c**) mice treated with ethanolic extract of *E. guttatum* and (**d**) mice treated with methanolic extract of *E. guttatum*.

The pancreas of normal mice showed pancreatic acini (PA) with normal histological appearance and normal inter-acini and interlobular spaces (Figure 3a). Pancreas histology of mice treated with the aqueous extract (200 mg/kg) showed only intra-pancreatic inflammation (Figure 3b). Additionally, pancreas sections from mice treated with the ethanolic extract showed only minimal focal intralobular inflammation (Figure 3c), while the pancreas of mice treated with the methanolic extract showed intra-pancreatic inflammation and increased plasma cells (Figure 3d).

## 3. Discussion

Currently, medicinal plants are known for their pharmacological effects. However, less is known about the potential toxicity of their biologically active substances [16,17]. A study of acute toxicity examines the adverse effects that occur in the short term after the administration of a single dose of a tested product. These tests are generally conducted on rodents and are carried out at the beginning of the development of a new substance in order to provide information on its toxicity [18]. The present study showed that aqueous, ethanolic and methanolic extracts of *E. guttatum* did not cause death or behavioral changes in mice at a dose of 2000 mg/kg. According to the OECD classification, with the LD_50_ being superior to 2 g/kg, this plant can be considered as not presenting a risk of acute toxicity.

Our study was conducted to evaluate the subacute toxicity of *E. guttatum* L. extracts during a 28-day experiment in mice. Subacute toxicity allows for the examination of the cumulative toxicity of a substance in the target organs or the physiological and metabolic effects of the compound by prolonged exposure to low dosage. A wide variety of adverse effects can be detected from subacute toxicity studies, and the long-term safety of a compound can be predicted. In our study, the results showed that mice treated with *E. guttatum* extracts at a dose of 200 mg/kg showed no signs of toxicity, and no deaths were recorded.

Changes in body weight have been used as an important indicator to assess the adverse effects of drugs and chemicals [19,20]. In our study, there was a progressive, normal increase in the mean body weights of both the treated and control groups. In addition, the difference in weight gain between the controls and the groups treated with the extracts at the dose of 200 mg/kg was statistically insignificant. According to Raina et al. [21], organ weights are markers of the state of pathological and physiological well-being of the animals. When herbal products are ingested, they can be toxic to vital organs such as the kidneys, liver and pancreas due to their various roles in the human body. Based on the organ weight results of the three treated groups compared to the control group, there were no significant weight changes in the liver, kidney or pancreas.

According to Wu et al. [22], anemia following the administration of a product may cause hemolysis and/or inhibition of hematopoiesis, as well as a decrease in hematological parameters due to the bioactive components of the extract. Blood is the main vehicle responsible for the distribution of nutrients and foreign substances in the body. Therefore, the constituents of blood, namely erythrocytes, leukocytes, platelets and hemoglobin are exposed to high doses of toxins [23,24]. Subacute administration of *E. guttatum* extracts resulted in an increase in white blood cells, platelets, lymphocytes, monocytes, basophils and neutrophils in all three groups. These results suggest that *E. guttatum* contains bioactive molecules that have an amplifying effect on the immune response by increasing the number of white blood cells [25]. The results did not show a significant change in red blood cell and hemoglobin levels, which indicates that the extracts may not contain toxic substances that can cause anemia or other abnormalities.

Biochemical analysis showed a slight, non-significant increase in total cholesterol, triglycerides, LDL and HDL levels. These results suggest that *E. guttatum* may induce the secretion of total cholesterol, LDL and HDL from the liver. In addition, ASAT and ALAT levels at 200 mg/kg were slightly elevated in mice treated with the methanolic and ethanolic extracts, while mice treated with the aqueous extract were able to decrease ASAT levels compared with the control, but this was not significant. An abnormal elevation of liver enzymes (ALAT and ASAT) is related to liver damage or impaired bile flow. ALAT (alanine aminotransferase) is a liver-specific enzyme that is released into the blood when a cell is damaged or injured. ASAT (aspartate aminotransferase) is an enzyme found mainly in red blood cells, heart and skeletal muscle and the kidneys [26,27]. The liver is the site of elimination or degradation of cholesterol and the main site of synthesis. The fact that a slight significant change was observed in cholesterol and triglyceride levels in this study suggests that *E. guttatum* may not have side effects on cholesterol metabolism in mice. The study carried out on the aerial part of *E. guttatum* showed that, at high doses (2000 mg/kg and 5000 mg/kg), the aqueous extract did not cause any change in the levels of biochemical parameters, such as ASAT, ALAT, urea, creatinine, cholesterol, triglycerides and blood glucose compared to the control group [28]. This confirms that the aqueous extract has hepatoprotective and nephroprotective effects.

Renal function can be assessed by changes in creatinine, urea and glucose, and increases in these parameters indicate injury and impairment of the renal filtration mechanism [29,30]. In our study, the average creatinine level was slightly increased in mice treated with methanolic and ethanolic extracts. On the other hand, the creatinine level in mice treated with the aqueous extract remained within normal ranges. The mean values of urea and glucose were low in the three treated groups compared to the control. These results suggest that *E. guttatum* has no negative effect but seems to have a protective effect on the kidney. Based on these results, it is reasonable to assume that repeated administration for 28 days may cause toxicity to vital organs.

Histopathological studies serve as supportive evidence for hematological and biochemical analyses [31]. The photomicrographs of sections of the liver, kidney and pancreas of mice treated orally with extracts of *E. guttatum* at a dose of 200 mg/kg for 28 days showed histological changes such as an intralobular mononuclear inflammatory focus, a mononuclear inflammatory focus around the centrilobular veins (CLV), hydropic cytoplasmic degeneration of hepatocytes, binucleate cells and a nucleus, a low-bulk, dark-pigment glomerular lesion, interstitial inflammation, plasma cells in the interstitium, and intrapancreatic inflammation. Therefore, these histopathological findings corroborate the biochemical results, and a chronic study is necessary for a complete understanding of the hepatoxicity of this plant.

## 4. Conclusions

Our preliminary study evaluated the acute and subacute safety or toxicity of aqueous, ethanolic and methanolic extracts of *E. guttatum* after oral administration in mice. The acute toxicity study showed that the LD_50_ value was greater than 2000 mg/kg, and the extracts showed no signs of toxicity during the 14 days of the study. In the subacute toxicity study, no deaths were recorded after oral administration of 200 mg/kg of *E. guttatum* for 28 days. The mice showed liver toxicity, particularly those treated with the methanolic extract, as determined by hematological, serum biochemical and/or histological analyses. These results provide preliminary information on the toxicity of *E. guttatum*; thus, these results show the potential cytotoxic effects of methanol. Other evaluations, such as the toxicity of bioactive compounds and the neurotoxicity, reprotoxicity and genotoxicity of this plant, should be performed in future studies. Finally, it is necessary to assess the safety and toxicological aspects of all other traditionally used medicinal plants.

## 5. Materials and Methods

### 5.1. Plant Material and Extraction

The roots of *E. guttatum* were collected from the Oujda Region, Morocco. The specimen was identified by a botanist, Mohammed Fennane, and was deposited to the Botanical Herbarium of the Scientific Institute of Rabat under the reference number RAB 110970. The root parts were dried in the dark at room temperature. The plant material was then reduced to powder and subsequently used for the preparation of the extracts.

The plant material was prepared by utilizing two types of extraction methods (infusion and maceration). The aqueous extract was prepared using the infusion method, by which 50 g of *E. guttatum* powder was infused with 500 mL of distilled water for 1 h and then left to cool. The extract was filtered and evaporated at 50 °C using a rotary evaporator. Subsequently, the extract was freeze-dried and stored until use. For the ethanolic and methanolic extracts, 50 g of the root powder was macerated with 500 mL of ethanol and methanol, respectively, and stirred for 48 h at room temperature. The extract was filtered through Whatman n1 paper and evaporated at 40 °C using a rotary evaporator. The extracts obtained were stored at a temperature of 4 °C.

### 5.2. Experimental Animals

Female and male Swiss albino mice weighing between 25 to 35 g were used in this study. The animals were kept at an ambient temperature of 25 °C and a light/dark cycle of 12 h in cages at the Faculty of Medicine and Pharmacy in Rabat. They had free access to water and normal food throughout the experiment. The experiment was carried out according to the principles described in the "Guide to the care and use of laboratory animals," 8th edition, prepared by the National Academy of Sciences (National Research Council of the National Academies). Every effort was made to minimize animal suffering and the number of animals used for the study. Ethics approval was obtained from Mohammed V University in Rabat.

### 5.3. Acute Oral Toxicity

Acute oral toxicity was carried out according to the methods described in the guidelines of the Organization for Economic Co-operation and Development, No. 423 (OECD 423) [5]. The toxicity of *E. guttatum* extracts was evaluated in female Swiss mice. The animals were divided into four groups of six mice each.

Group I (Control): animals were administered vehicle (distilled water) orally.

Group II, III and IV: animals were administered 2000 mg/kg of their body weight with either aqueous, ethanolic or methanol extract of *E. guttatum* root, respectively, via oral gavage.

After the administration of a single dose of the different extracts of *E. guttatum*, the animals were observed for 14 days in order to note the behavior of the animal, the signs of possible toxicity and the number of deaths due to the extracts.

### 5.4. Sub-Acute Oral Toxicity

The subacute oral toxicity study was conducted in accordance with the guidelines of the Organization for Economic Co-operation and Development (OECD 407) [6]. Thirty-two animals were randomly assigned to four groups of eight animals each. Group I, control, received distilled water (vehicle). Groups II, III and IV received an oral dose of 200 mg/kg of either the aqueous, ethanolic or methanolic extracts of *E. guttatum*, respectively, and was administered once daily for 28 consecutive days. Body weights were recorded once a week throughout the study period.

#### 5.4.1. Determination of Hematological and Biochemical Parameters

At the end of the experimental period, blood samples were taken from the jugular vein using tubes in heparinized tubes for hematological studies and non-heparinized tubes from which serum was isolated by centrifugation at 3000 rpm for 10 min and then used for biochemical assessments.

A complete blood count (CBC) and differential were performed on the blood samples using Sysmex KX21N, an automated 3-part differential hematology analyzer (Sysmex Corporation Kobe, Japan). Standardization, calibration of the instrument, and processing of the samples were performed according to the manufacturer’s instructions. The machine automatically dilutes whole-blood samples to 50 mL in the CBC/Differential mode and then lyses and enumerates white blood cells (WBC), red blood cells (RBC), hemoglobin concentration (Hb) and platelets. However, it does not count for eosinophils, monocytes or basophils. Therefore, a manual differential count was conducted on well-prepared thin-blood films colored using the May Grünwald Giemsa (MGG) method. The parameters studied under the optical microscope were: (1) The RBC morphology to detect possible corpuscular anomalies. (2) The leucocyte parameter was determined, double-blinded, by two operators. Each of them established the percentage of the different leucocyte populations (NEU, EOS, BAS, MONO and LYM) on 200 leucocyte elements. In case of differences of more than five cells for a leucocyte population, formulas were double-checked by two additional readings (by the same operators). The final formula was calculated from the average of both formulas. The values obtained from the NEU, EOS, BAS, LYM and MONO, expressed in 109/L, were deducted from the leucocyte numeration measured by the automation. (3) Platelets study was conducted to research morphological anomalies or platelet aggregates.

Serum levels of aspartate aminotransferase (ASAT), alanine aminotransferase (ALAT), urea, creatinine, cholesterol, triacylglycerols (TG), high-density lipoproteins (HDL) and low-density lipoproteins (LDL) were determined using an Abbott Architect c8000 model, Clinical Chemistry System, USA, auto-analyzer according to manufacturer instructions.

#### 5.4.2. Organ Weights and Histopathology

Animals were sacrificed under mild ether anesthesia. After the sacrifice, the weights of the organs (liver, kidney and pancreas) were recorded. Vital organs were excised from the anesthetized animal and rinsed in 0.9% saline solution. Tissue pieces were fixed in 10% paraformaldehyde for paraffin histology and processed in paraffin embedding according to standard protocol. Sections of each tissue were stained with hematoxylin and eosin and observed for possible histopathological damage.

### 5.5. Statistical Analysis

Data were expressed as means ± SD. Statistical analyses and the comparison of means were evaluated using ANOVA (Tukey’s test). The differences were considered statistically significant at *p* < 0.05. Analyses were performed with GraphPad Prism 6 (San Diego, CA, USA).

## Figures and Tables

**Table 1:** Effects of acute oral administration of aqueous and alcoholic extracts of *E. guttatum* on the body weights of Swiss mice with a dose of 2000 mg/kg.

Extracts	Extract Dose mg/kg	Body Weight (g)		
		Initial Weight (1st Day)	Final Weight (14th Day)	Difference
Aqueous extract	2000	31.67 ± 3.22	32.53 ± 0.49	+0.86
Ethanolic extract	2000	23.41 ± 0.57	25.21 ± 1.30 *	+1.8
Methanolic extract	2000	28.10 ± 1.56	28.54 ± 2.71	+0.44
Control group	Distilled water	27.82 ± 4.78	30.63 ± 2.20	+2.81

Data are expressed as mean ± SD (*n* = 6). * Significantly different from the control group, *p* < 0.05.

**Table 2:** Effects of subacute oral administration of aqueous and alcoholic extracts of *E. guttatum* on the body weights of Swiss mice with a dose of 200 mg/kg.

Extracts	Extract Dose mg/kg	Body Weights (g)		
		Initial Weight (1st Day)	Final Weight (28th Day)	Difference
Aqueous extract	200	29.5 ± 1.67	31.47 ± 2.74	+1.97
Ethanolic extract	200	30.12 ± 1.41	32.19 ± 2.15	+2.07
Methanolic extract	200	27.83 ± 2.66	29.6 ± 2.01	+1.77
Control group	Distilled water	25.75 ± 1.18	27.43 ± 1.32	+1.68

Data are expressed as mean ± SD (*n* = 8). *p* > 0.05, not significant.

**Table 3:** Effect of subacute oral administration of aqueous and alcoholic extracts of *E. guttatum* on organ weights in Swiss albino mice.

	Control	Aqueous Extract	Ethanol Extract	Methanol Extract
Liver	1.63 ± 0.09	1.62 ± 0.13	1.46 ± 0.21	1.63 ± 0.32
Kidney	0.37 ± 0.04	0.43 ± 0.09	0.35 ± 0.12	0.39 ± 0.10
Pancreas	0.098 ± 0.06	0.092 ± 0.02	0.072 ± 0.02	0.093 ± 0.02

Data are expressed as mean ± SD (*n* = 8). *p* > 0.05, not significant.

**Table 4:** Effects of subacute oral administration of aqueous and alcoholic extracts of *E. guttatum* on hematological parameters in Swiss albino mice.

	Control	Aqueous Extract	Ethanol Extract	Methanol Extract
**WBC 10^3^/μL**	4.18 ± 0.34	11.26 ± 4.33 *	8.05 ± 0.36	7.92 ± 1.77
**RBC 10^6^/μL**	8.42 ± 1.01	8.81 ± 1.01	8.36 ± 1.58	8.14 ± 0.78
**HGB g/L**	12 ± 1.55	12.82 ± 1.33	12.63 ± 2.22	11.82 ± 1.52
**PLT 10^3^/μL**	944 ± 11.33	1034 ± 316.4	1030.3 ± 244.3	1097.7 ± 182.5
**LYMPH 10^3^/μL**	3.66 ± 0.39	9.32 ± 3.88	5.81 ± 0.78	6.22 ± 1.72
**MONO 10^3^/μL**	0.025 ± 0.02	0.155 ± 0.02	0.12 ± 0.028	0.195 ± 0.12
**BASO 10^3^/μL**	0.01 ± 0.00	0.035 ± 0.03	0.013 ± 0.005	0.025 ± 0.01
**NEUT 10^3^/μL**	0.48 ± 0.02	1.72 ± 0.74	2.08 ± 1.11	1.47 ± 0.52

WBC: White blood cell, RBC: Red blood cell, HGB: Hemoglobin; PLT: Platelet, LYMPH: Lymphocyte, MONO: Monocyte, BASO: Basophil, NEUT; Neutrophil. Data are expressed as mean ± SD (*n* = 8). * Significantly different from the control group, *p* < 0.05.

**Table 5:** Effects of subacute oral administration of aqueous and alcoholic extracts of *E. guttatum* on biochemical parameters in Swiss mice.

	Control	Aqueous Extract	Ethanol Extract	Methanol Extract
CHOL.T (G/L)	0.94 ± 0.17	1.30 ± 0.31	1.01 ± 0.25	0.94 ± 0.29
TRIG (G/L)	0.91 ± 0.13	1.1 ± 0.20	0.81 ± 0.32	0.99 ± 0.28
LDL (G/L)	0.18 ± 0.14	0.34 ± 0.19	0.27 ± 0.14	0.23 ± 0.09 *
HDL (MMOL/L)	0.58 ± 0.06	0.74 ± 0.14	0.57 ± 0.16	0.51 ± 0.24
ASAT (UI/L)	183.33 ± 72.1	169 ± 39.2	193 ± 23.3	238.25 ± 37.3 *
ALAT (UI/L)	32.33 ± 9.07	32.25 ± 3.5	40.33 ± 23.5	73.25 ± 49.3
CREAT (MG/L)	4.00 ± 1.00	3.75 ± 0.95	4.33 ± 0.57	4.5 ± 0.57
UREA (G/L)	0.273 ± 0.05	0.18 ± 0.03	0.21 ± 0.06	0.22 ± 0.06
GLU (G/L)	1.51 ± 0.23	1.49 ± 0.17	1.13 ± 0.14	1.06 ± 0.23

Data are expressed as mean ± SD (*n* = 8). * Significantly different from the control group, *p* < 0.05.

## Data Availability

The data presented in this study are available in this article.
